# Bringing pathology to nanomedicine: a comparison of in vivo toxicity of polymeric nanoparticle carriers with and without chitosan coating

**DOI:** 10.1007/s00428-023-03581-y

**Published:** 2023-07-04

**Authors:** Christos Poulios, Varvara Karagkiozaki, Dorothea Kapoukranidou, Zena Chakim, Thomas Zarampoukas, Nikolaos Foroglou, Stergios Logothetidis

**Affiliations:** 1 Department of Pathology, Faculty of Medicine, Aristotle University of Thessaloniki, Thessaloniki, Greece; 2 European Society of Pathology, Brussels, Belgium; 3 Laboratory of Thin Films, Nanobiomaterials–Nanosystems and Nanometrology, Faculty of Physics, Aristotle University of Thessaloniki, Thessaloniki, Greece; 4 BL NanoBiomed, Thessaloniki, Greece; 5 Department of Physiology, Faculty of Medicine, Aristotle University of Thessaloniki, Thessaloniki, Greece; 6 Department of Neurosurgery, AHEPA University Hospital, Aristotle University of Thessaloniki, Thessaloniki, Greece

**Keywords:** PLGA, Animal model, Wistar rat, Curcumin, Nanotechnology, Neurotoxicity

## Abstract

Over the last years, there has been an increasing number of proposals for the use of nanomaterials in medicine. The safety of novel technologies must be verified, prior to their clinical application. Pathology has much to contribute towards this end. In this study, we compared the in vivo toxicity effects of poly- (lactic-co-glycolic acid) nanoparticles with and without chitosan shell. Both nanoparticle types were loaded with curcumin. The nanoparticles were assessed in vitro for potential cytotoxicity with cell viability studies. For the in vivo test, 36 adult Wistar rats were used, four of which were the control group. The remaining 32 were divided into 2 groups, each of which was administered differentially coated drug carriers: (A) nanoparticles without chitosan coating and (B) nanoparticles with chitosan coating. For both groups, the subcutaneous route was used for administration. Each group was further divided into 2 sub-groups of 8 animals each. The animals of the first sub-groups were sacrificed 24 h after the injection and those of the second on the 7th day. The control group was also divided into 2 subgroups of 2 animals each. At the appointed post-administrative date, the rats were sacrificed, and specimens from the brain, liver, kidneys, heart, stomach, lungs, and from the skin at the injection site were collected and studied histopathologically. The evaluation of both in vitro and in vivo testing shows that nanoparticles with chitosan have significantly less, if any, toxic effects compared to those without chitosan.

## Introduction

Pathology is the bridge between Science and Medicine. No other medical specialty can provide information for the basis of disease and assess its effects from the macroscopical down to the molecular level.

The introduction of nanomaterials has expanded the boundaries of Medicine. The roles of nanoparticles (NPs) in Medicine include, among others, the ability to be used as drug carriers. NPs can provide increased bioavailability [1] and have high efficiency [2]. These properties, especially for biodegradable NPs, mean that a drug can be administrated in a lower dose and still be equally, if not more, effective than usual drugs while reducing the risk of toxic adverse effects. NPs have recently gained the attention of the public, as lipid NPs were used as carriers for the newly developed vaccines for coronavirus disease 2019 (COVID-19) [3].

Due to their nature, NPs can deliver in aquatic solutions drugs that are hydrophobic and could not be otherwise administrated by injection. One example for such drug is curcumin, which is a polyphenol compound, extracted from the root of *Curcuma longa* (turmeric). Chemically curcumin is a 1,7-bis(4-hydroxy-3-methoxyphenyl)-1,6-heptadiene-3,5-dione, commonly called diferuloylmethane. It has been gaining a lot of attention over the last years, mainly for its antioxidant and anti-inflammatory attributes. Curcumin has been proposed as a treatment for various conditions including Alzheimer’s disease, hypercholesterolemia, atherosclerosis, psoriasis, Crohn’s disease, neurological disorders, and cancer [2]. The great pharmacological potential of curcumin is hampered by its electrochemical properties [1] including among others its hydrophobic nature and non-solubility in water [4]. Therefore, a development of a drug-carrier that can efficiently and safely transport curcumin in an aqueous solution is a big step for its clinical implementation.

Poly lactic-*co*-glycolic acid (PLGA) (linear formula: [C_3_H_4_O_2_]x[C_2_H_2_O_2_]y) is a copolymer of poly lactic acid (PLA) and poly glycolic acid (PGA) [5]. PLGA is biodegradable in water solutions. It has been used for many medical products, ranging from material for absorbable sutures to drug carrier, that have received US FDA approval [6]. The medical applications thus far include the use of PLGA either as bulk material or in microparticle scale. Microparticles measure between 1 and 1000 μm, whereas nanoparticles have a diameter less than 1 μm [6]. Over the last years, there is an increased interest for the medical application of PLGA on nanoscale level, to achieve the benefits of NPs as mentioned above.

Chitosan is a polysaccharide polymer produced by chitin and is one of the most exploited polymers in biomedical science. Chitosan is polycationic, that makes it water soluble and bioadhesive that readily binds to negatively charged surfaces such as mucosal membranes. Chitosan biodegrades into harmless products (amino sugars) [7]. Considering the above, chitosan is an excellent material to be used for the construction of drug-carriers, either on its own or combined with other materials.

Although in vitro and cell culture toxicity studies are a fundamental step in the assessment of potential toxic effects, our current means do not offer complete simulation of the complicated biochemical and cellular conditions of the human body. Thus, in vivo testing is the next step for toxicity studies.

The scope of this study is to compare the potential in vivo toxicity of PLGA NPs that carry curcumin with and without chitosan coating, by utilizing simple methodology, available in any Pathology laboratory.

## Materials and methods

The synthesis on the NPs, their characterization, and the in vitro studies were performed at the Laboratory of Thin Films, Nanobiomaterials–Nanosystems and Nanometrology (LTFN), Faculty of Physics, Aristotle University of Thessaloniki (AUTH). The animal testing was performed at the Laboratory of Physiology, Faculty of Medicine, AUTH. The histopathological examination was performed at the Pathology Department, Faculty of Medicine, AUTH.

### NP synthesis and characterization

All synthesis and characterization experiments were conducted in sterile conditions to avoid contamination. For the synthesis of the NPs, we used nanoprecipitation. This method is used for the synthesis of similar NPs [8]. First, PLGA [lactide:glycolide (75:25), molecular weight 66,000–107,000, code P1941, Sigma-Adrich] was diluted in acetone and left on vortex overnight. In the solution, acetone-diluted curcumin (molecular weight 368.38, code C1386 Sigma-Aldrich) was then added with a ratio polymer/medicine: 9/1. As curcumin is sensitive to light, all procedures were carried out either in low-light conditions and, when possible, with the various containers covered by tin foil.

This solution was the organic phase, which was added in drop-wise manner to the aqueous phase (polyvinyl alcohol, PVA, 1%) under stirring. The organic:aqueous ratio was 1:2. The combined solution was left overnight for the solvent to evaporate and was then centrifuged for aggregations to be removed. At this point, for the NPs that were coated with chitosan, an acetic acid solution of chitosan (low molecular weight, code 448869, Sigma-Adrich) was added in a ratio NPs/chitosan: 3/1. The solutions for both types of NPs, were centrifuged and filtered. In the final solution, the concentration of NPs was 10 mg/ml.

**Fig. 1:**
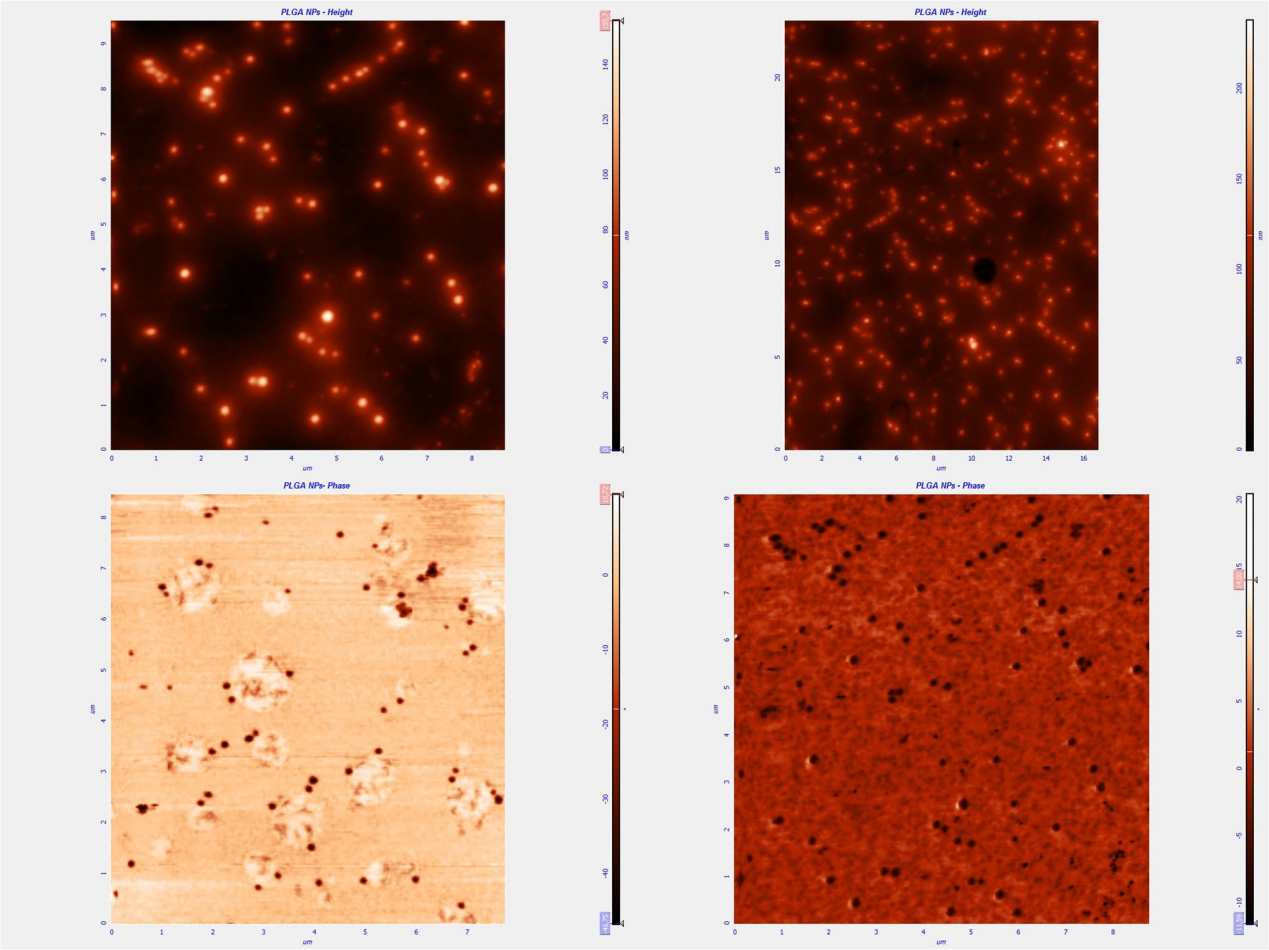
Atomic force microscopy (AFM) morphological characterization of nanoparticles. Height and phase images for NPs with chitosan (left column) and without (right column)

The final product was characterized morphologically by means of atomic force microscopy (AFM) study (Fig. 1). The mean diameter of NPs without chitosan ranged from 140 to 220 nm and from 166 to 218 nm for NPs with chitosan. Biodegradation and drug release studies were performed, as part of other LTFN projects [9, 10]. The results of those studies showed that drug uptake was around 85% and in the final solution the concentration of curcumin was 1 mg/ml. Moreover, in conditions simulating those in the human body, about 50% of the drug was released within the first 2 days, and this increased to 90% by the fifth day. After 3 weeks (21–25 days), 95% of the NPs had degraded. The findings were similar for both types of NPs, without chitosan/plain (NPP) and with chitosan (NPC).

### Cell viability studies

To evaluate potential cytotoxic effects and to compare any differences between NPs with and without chitosan, a MTT test was performed. The methodology was similar as in other LTFN projects [9, 10]. In brief, this test includes the cultivation in wells that contain NPs, cells of the L929 line. In each well, MTT (thiazolyl blue tetrazolium bromide), which is a yellow dye, is added. This dye is reduced to purple-colored formazan in living cells. The light absorbance from well samples was measured by spectrophotometry at 560-nm wavelength, on days 1, 2, and 3 and compared to this of the control. The higher the absorbance, the more viable cells each well contained. In our protocol, viability above 80% was an acceptable result for biocompatibility, and viability comparable to or higher than the control was considered as excellent.

### In vivo toxicity studies

This study was designed taking into consideration the three Rs of animal testing: Replacement, Reduction, and Refinement.

As test animal, the Wistar rat was selected (*Rattus norvegicus*), not only for the easy handling but also as this animal is used in similar studies [11]. As preliminary results from in vitro testing [9, 10] were encouraging, and considering the published literature [1, 11], we did not perform a lethal dose (LD)50 test, to drastically reduce the number of animals used. For the calculation of the sizes of the groups of animals to be used, we used the G* Power 3.1 software [12] and compared the software results with the bibliography for similar projects.

#### Animal selection and drug administration

A total of 36 adult Wistar rats were selected, with equal number of males and females. Thirty-two rats were divided into two groups of 16 animals, one group to receive NPPs and the other NPCs and caged in controlled conditions with a cycle of 12-h light and 12-h darkness. Food and water were provided freely. The animals were acclimated to their environment for 1 week prior to the testing.

Using the data from the drug-release studies and considering the higher body temperature and metabolic rate of rats [13], it was decided for half of the animals to be sacrificed 24 h after the administration of the drug and the other half 1 week after the administration. The weight of the animals was measured both on the day of the injection and on the day of sacrifice.

As control, we used 4 animals, 2 to be sacrificed 24 h after injection and 2 1 week later. The control group received an injection of phosphate-buffered saline (PBS) solution, to evaluate if there are any adverse effects caused by the injection itself and not the NPs. A relative low number of animals for the control group was decided in compliance to the three R principle, as the findings from the control group were expected to be similar if not identical to those in normal tissue.

All animals received a single subcutaneous injection. The dose of drug each rat received was 25 ml per kilogram (Kg) of body weight.

#### Animal sacrifice

At the appointed post-administration date, the animals were sacrificed. Initially, they were sedated with endoperitoneal injection of ketamine (Imalgene; Merial S.A, France; 50 mg/kg) and xylazine (Sedaxylan; Eurovet Animal Health B.V., Handelsweg, The Netherlands; 5 mg/kg) mixture. To ensure tissue fixation with minimal cold ischemia time, formalin (10%, neutral buffered) was administrated in the blood circulation with perfusion, prior to the extraction of the organs.

#### Histopathological studies

After the sacrifice, the brain, heart, lungs, stomach, kidneys, liver, and a section from the skin at the injection area were extracted. The brain was sectioned coronally and was sampled completely. The heart, stomach, and kidneys were also sampled completely. Three random sections were sampled from the liver and one from each lung. All tissue samples were fixed in formalin for 24 h and were embedded in paraffin. Sections 3–5 μm (micrometers) thick were stained with hematoxylin-eosin (H/E).

The histopathological examination was the main focus of the present study, and its goal was to examine if there were any toxicity-related lesions in the samples from the test animals.

The increase in the number of inflammatory cells was marked in a quantitative way. This was done in a 4-grade system (0–3), 0 for no increase compared to normal histology, 1 for mild, 2 for moderate, and 3 for significant increase. Similar approaches are being used in Pathology diagnostic practice, for example, in the assessment of inflammation in gastritis [14].

The second goal was to check for tissue injury. This may be either reversible or irreversible [15]. As any sign of cell injury is a severe indication for toxicity, reversible injury was graded as 2 and irreversible as 3, following the inflammatory grading system. The scores of inflammation and injury were added for each examined organ of each animal. Thus, each organ received a total score with a maximum range from 0 to 6.

Any other finding was recorded separately in a descriptive way.

#### Statistic analysis

The results of all 4 subgroups were compared to those of the control group. To determine if any potential difference between the scores of each group compared to the score of the control was statistically significant, a two-tail Student’s *t*-test was performed. Results with *P* value < 0.05 were considered as statistically significant.

## Results

### Results of the cell toxicity studies

**Graph 1:**
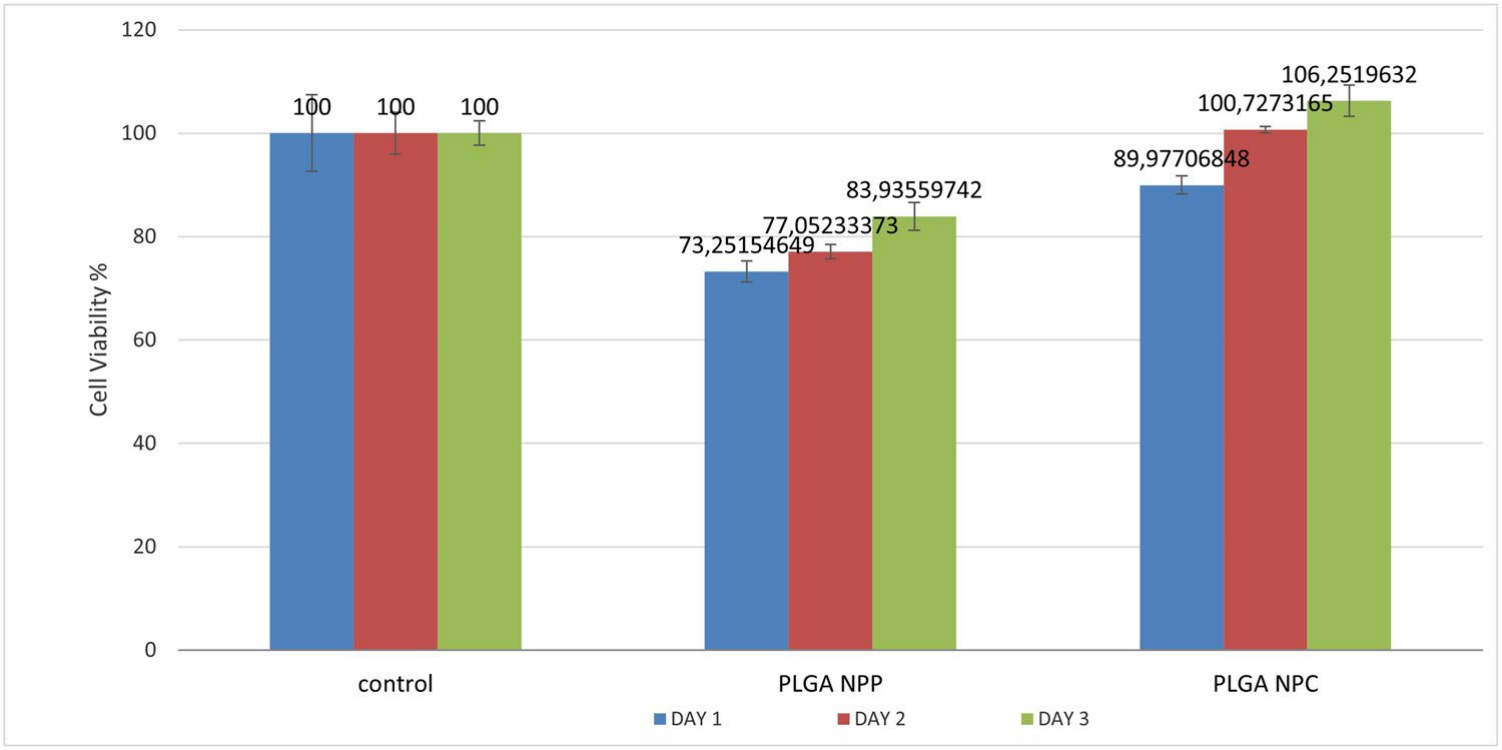
Results from the MTT test. NPP, PLGA nanoparticles without chitosan. NPC, PLGA nanoparticles with chitosan. This is a graphic representation of the results from Table 1

The results of the MTT test are presented in Table 1 and Graph 1. The wells that received NPPs had reduced cell viability that for days 1 and 2 was below 80% and on day 3 above 80% when compared to the control wells. The wells that received NPCs had 90% cell viability on the first day and above 100% for days 2 and 3. The last finding means that in the wells with NPCs had more viable cells than the control wells.

**Table 1:** Results from the MTT test. *NPP*, PLGA nanoparticles without chitosan; *NPC*, PLGA nanoparticles with chitosan. Wells with NPCs show higher percentage of viable cells compared to those with NPPs

		Control	PLGA NPP	PLGA NPC
Day 1	Cell viability	100	73.2515464867	89.9770684792
	stdev	7.4192251775	1.9976476461	1.3687925869
Day 2	Cell viability	100	77.0523337333	100.7273165437
	stdev	4.0262163146	1.3679383476	4.8805720938
Day 3	Cell viability	100	83.9355974176	106.251963163
	stdev	2.3724418444	2.67758641	2.8488195352

### Results of the in vivo studies

All animals survived to the scheduled day of sacrifice. No abnormal behavior, clinical signs of disease, or weight loss was observed for all groups. The results of the histopathological examination are presented in Table 2. It should be mentioned that both control subgroups showed normal histology for all examined specimens. Moreover, there was no significant difference between male and female animals.

**Table 2:** Detailed histopathological score of the examined organs from all animals. The total score for each animal was the number used in the statistic evaluation. The weight difference for all animals between administration and sacrifice days is listed. As all values are equal or above zero, no animal has lost weight. Explanation of animals coding: *C*, control; *NPC*, PLGA NPs with chitosan, with curcumin; *NPP*, PLGA NPs without chitosan, with curcumin; *A*, group sacrificed 1 day after administration; *B*, group sacrificed 1 week after administration. 1 to 8: animal number

Code	Sex	Animal weight difference in grams, between day of administration and day of sacrifice	Brain	Heart	Liver	Kidney	Lung	Stomach	Skin	Total score for all organs	Comments
CA1	Male	5	0	0	0	0	0	0	0	0	
CA2	Female	0	0	0	0	0	0	0	0	0	
NPPA1	Male	0	0	0	1	0	0	0	0	1	
NPPA2	Male	2	0	0	0	0	0	0	1	1	
NPPA3	Male	4	0	0	0	0	0	0	0	0	
NPPA4	Male	5	0	0	0	0	0	0	0	0	
NPPA5	Female	0	2	0	0	0	0	0	0	2	
NPPA6	Female	0	0	0	0	0	0	0	0	0	
NPPA7	Female	0	0	0	0	0	0	0	0	0	
NPPA8	Female	0	2	0	0	0	0	0	1	3	
NPCA1	Male	4	0	0	0	0	0	0	0	0	
NPCA2	Male	0	0	0	0	0	0	0	0	0	
NPCA3	Male	0	0	0	0	0	0	0	0	0	
NPCA4	Male	3	0	0	0	0	0	0	0	0	
NPCA5	Female	0	0	0	0	0	0	0	0	0	
NPCA6	Female	2	0	0	0	0	0	0	0	0	
NPCA7	Female	0	0	0	0	0	0	0	0	0	
NPCA8	Female	2	0	0	0	0	0	0	0	0	
CB1	Male	8	0	0	0	0	0	0	0	0	
CB2	Female	6	0	0	0	0	0	0	0	0	
NPPB1	Male	5	0	0	0	0	0	0	0	0	
NPPB2	Male	6	2	0	0	0	0	0	1	3	
NPPB3	Male	8	3	0	0	1	0	0	0	4	
NPPB4	Male	3	3	0	0	0	0	0	2	5	Cerebellum: calcifications
NPPB5	Female	4	3	0	0	0	0	0	0	3	
NPPB6	Female	5	3	0	1	0	0	0	2	6	
NPPB7	Female	3	0	0	0	0	0	0	1	1	
NPPB8	Female	5	4	0	0	0	0	0	1	5	Brain: two small areas with neurophagia; mild increase of inflammatory cells incl. rare neutrophils
NPCB1	Male	6	0	0	0	0	0	0	0	0	
NPCB2	Male	4	0	0	0	0	0	0	0	0	
NPCB3	Male	2	2	0	0	0	0	0	0	2	
NPCB4	Male	9	0	0	0	0	0	0	0	0	
NPCB5	Female	5	0	0	0	0	0	0	1	1	
NPCB6	Female	11	0	0	0	0	0	0	0	0	
NPCB7	Female	9	0	0	0	0	0	0	0	0	
NPCB8	Female	5	0	0	1	0	0	0	0	1	

#### Lungs, heart, stomach, liver, and kidneys

**Fig. 2:**
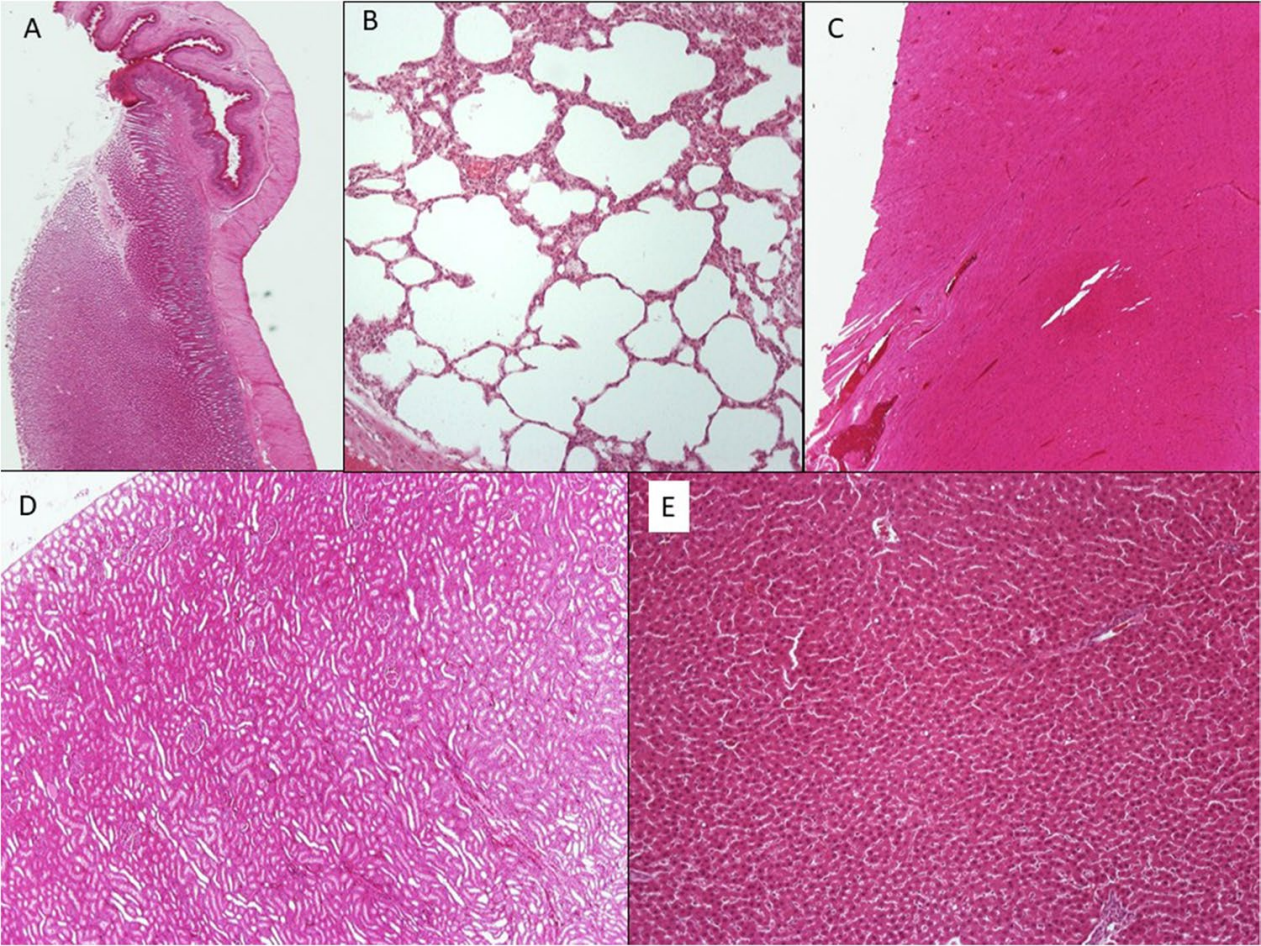
**A** H/E × 20, stomach, **B** H/E × 100, lung, **C** H/E × 40, kidney, **D** H/E × 40, heart, **E** H/E × 100, liver. The samples from the stomach, lungs, and heart from all examined animals did not show any significant findings. The samples from the liver and kidneys of most animals showed normal histology and only in four animals there was the incidental finding of focal mild inflammation

There were no significant findings for all animals in the examined samples from the lungs, heart, and stomach. The samples from the liver and kidneys also did not show areas of necrosis, and in only 4 animals, there was a mild, focal increase in inflammatory cells (Fig. 2).

#### Skin

**Fig. 3:**
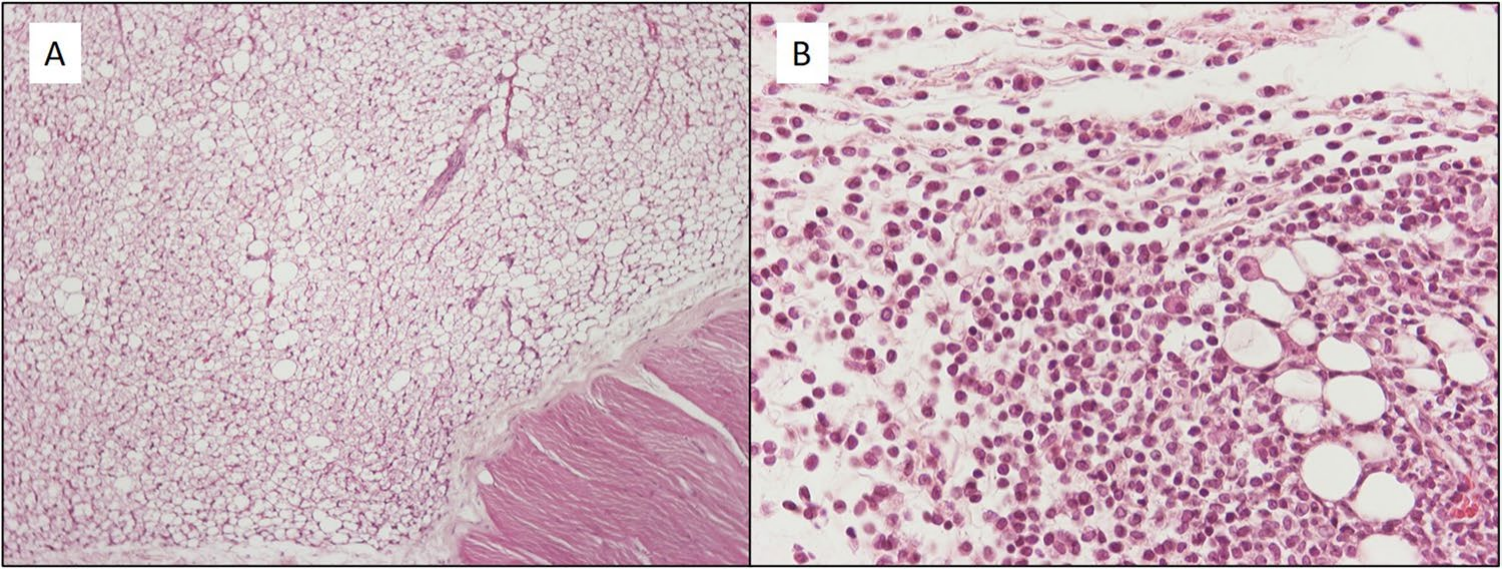
**A** H/E × 100, subcutaneous injection area of NPs with chitosan. No inflammation. **B** H/E × 400, subcutaneous injection area of NPs without chitosan. Mild increase of inflammatory cells, including lymphocytes, plasmacytes, macrophages, and neutrophils

The histological examination of samples taken from the injection area showed that 7 out of the 16 animals that received NPPs showed mild to moderate inflammatory reaction. For the animals that received NPCs, the number was 1 out of 16. The inflammatory cells were lymphocytes, plasmacytes, macrophages, and few neutrophils (Fig. 3).

#### Brain

**Fig. 4:**
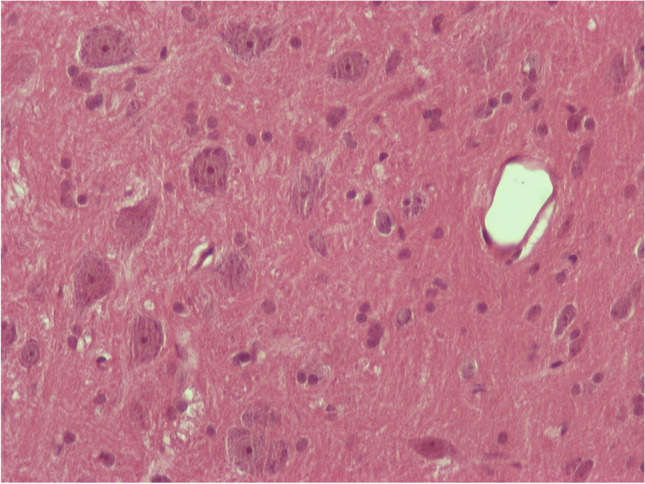
H/E × 400, brain sample from group receiving NPs with chitosan: normal findings

**Fig. 5:**
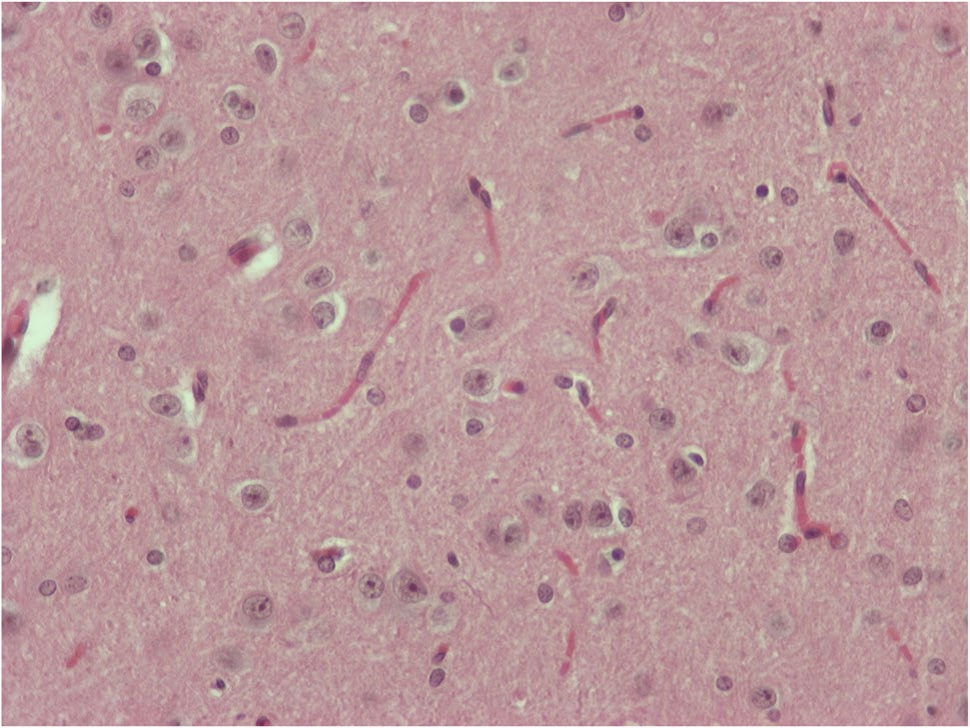
H/E × 400, brain sample from group receiving NPs with chitosan: pericellular and perivascular edema

For the animals that received NPCs and were sacrificed 24 h after the administration, there were no abnormal findings in the examined samples from the brain specimens (Fig. 4). Out of the 8 animals of the subgroup that was sacrificed 1 week after the administration, there was one animal that showed at the midbrain, at the area of the hypothalamus few glial cells, and rare neurons with mild pericellular edema, a reversible tissue damage. Perivascular edema was also observed in the same area (Fig. 5).

In the group that received NPPs, there were several animals that exhibited lesions in the samples from the brain. For the subgroup that was sacrificed 24 h after the administration, 2 out of the 8 animals showed at the areas of the hypothalamus and hippocampus focal pericellular edema.

In the subgroup that was sacrificed 1 week after the administration, six out of the eight animals showed lesions in the samples from the brain. One of the six showed at the areas of the hypothalamus and hippocampus pericellular and perivascular edema, receiving for the brain a score 2.

**Fig. 6:**
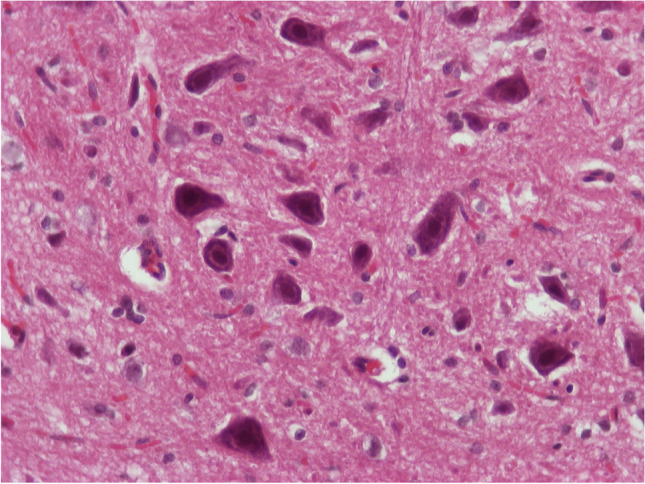
H/E × 400, brain sample from group receiving NPs without chitosan: “red neurons” and gliosis

**Fig. 7:**
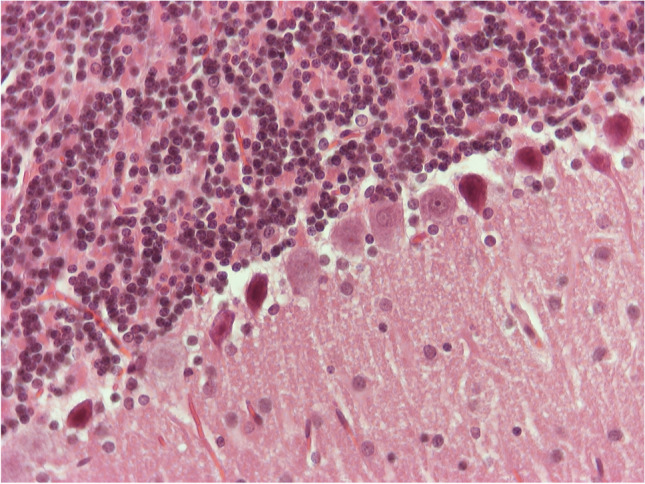
H/E × 400, cerebellum from group receiving NPs without chitosan: Purkinje cells with damage hyperplasia of Bergmann glia nearby

In all of the rest five animals, in addition to the previously mentioned edema, there were also observed:

A) In the areas of the hypothalamus and hippocampus: increased number of glial cells (gliosis) and many blood vessels with endothelial cells with increased size.
B) Several hippocampal neurons of pyramidal type, and many motor neurons at the midbrain with eosinophilic cytoplasm and distorted shape, the so-called “red neurons” (Fig. 6).
C) In the cerebellum, many Purkinje cells with damage (Fig. 7). Close-by, there was hyperplasia of Bergmann glia.

As these findings indicate irreversible damage and there was no inflammatory reaction observed, the score for the brain samples of those animals was 3.

**Fig. 8:**
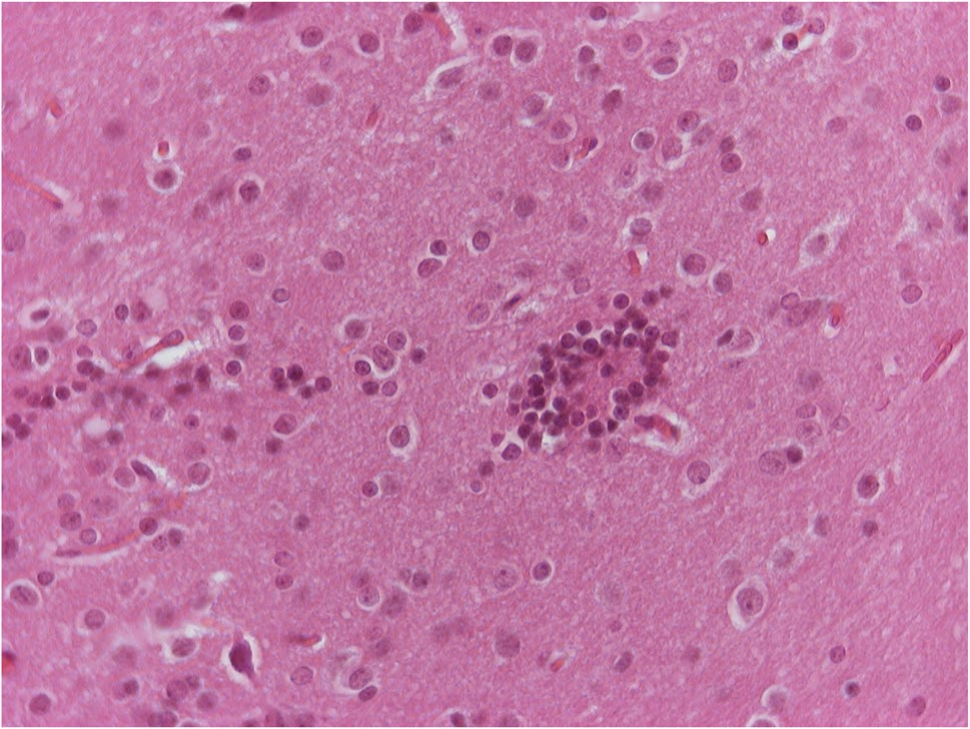
H/E × 400, brain from group receiving NPs without chitosan: area of neurophagia

**Fig. 9:**
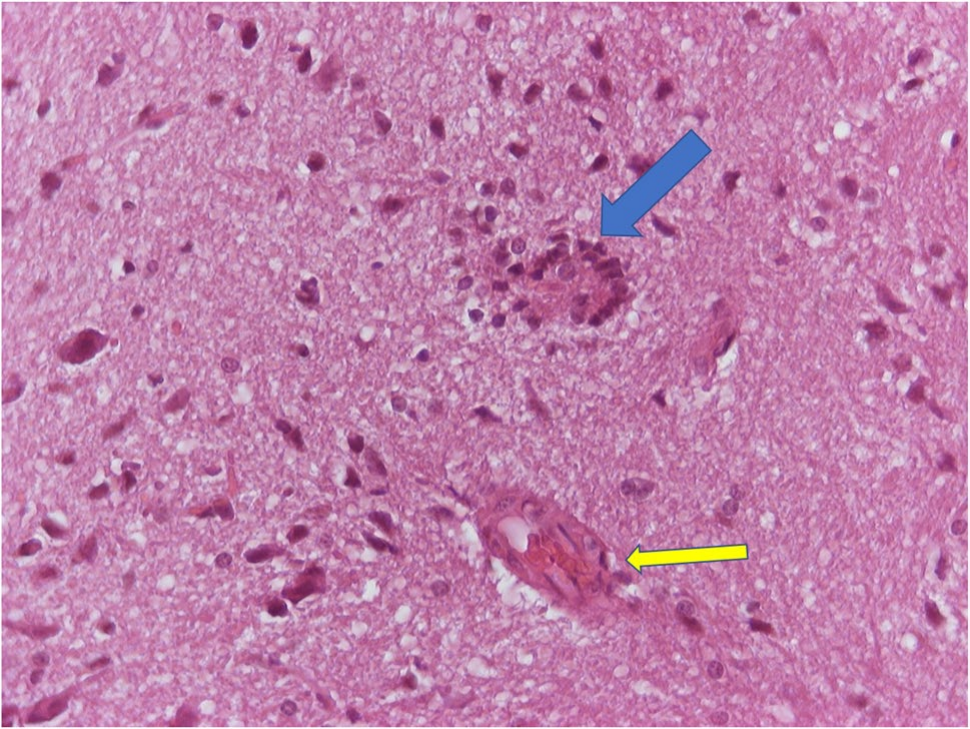
H/E × 400, brain from group receiving NPs without chitosan, same animal as in Fig. 8. Blue arrow: second area of neuronophagia. Yellow arrow: blood vessel with hyperplastic endothelial cells and perivascular edema

**Fig. 10:**
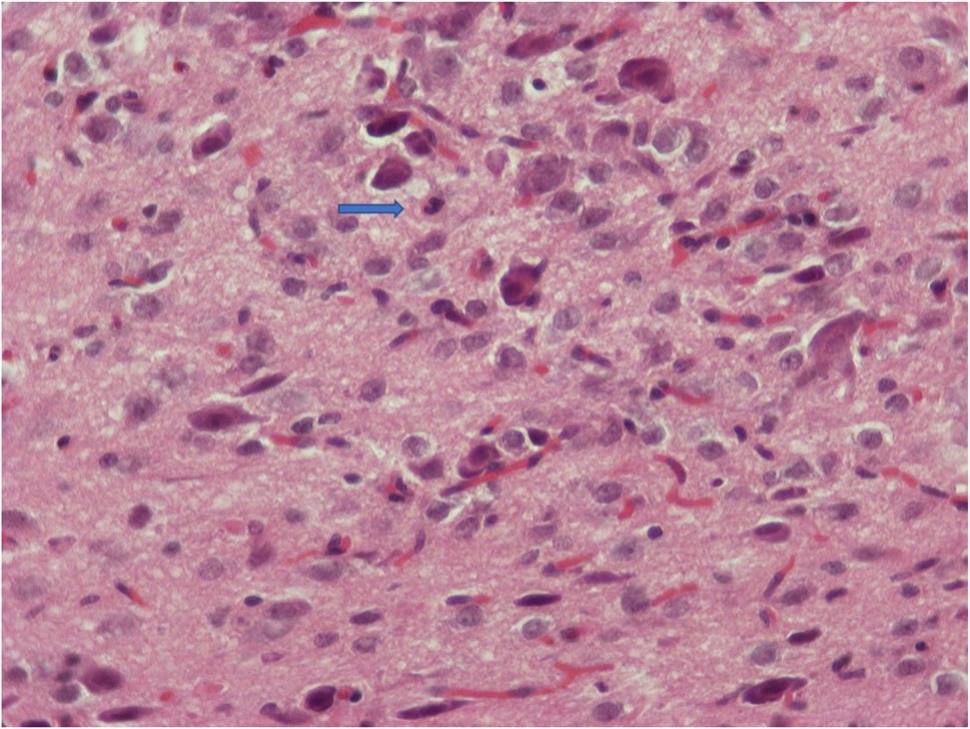
H/E × 400, brain from group receiving NPs without chitosan, same animal as in Figs. 8 and 9. Gliosis (hyperplasia/hypertrophia of glial cells), reactive endothelial cells, red neurons, and one neutrophil (arrow)

In one of those five animals, in addition to the above lesions, in the midbrain at the area of the deep mesencephalic nucleus, two small (0.9 mm and 1 mm in maximum diameter, respectively) areas of neurophagia were observed. The surrounding area showed not only gliosis and vessels with hyperplastic endothelial cells but also presence of rare neutrophils (Figs. 8, 9, and 10). Considering that in this animal there was not only irreversible damage but also mild inflammatory reaction, the score for the brain sample was 4. We need to emphasize that these findings were focal and easily to be overlooked or missed during sampling.

**Fig. 11:**
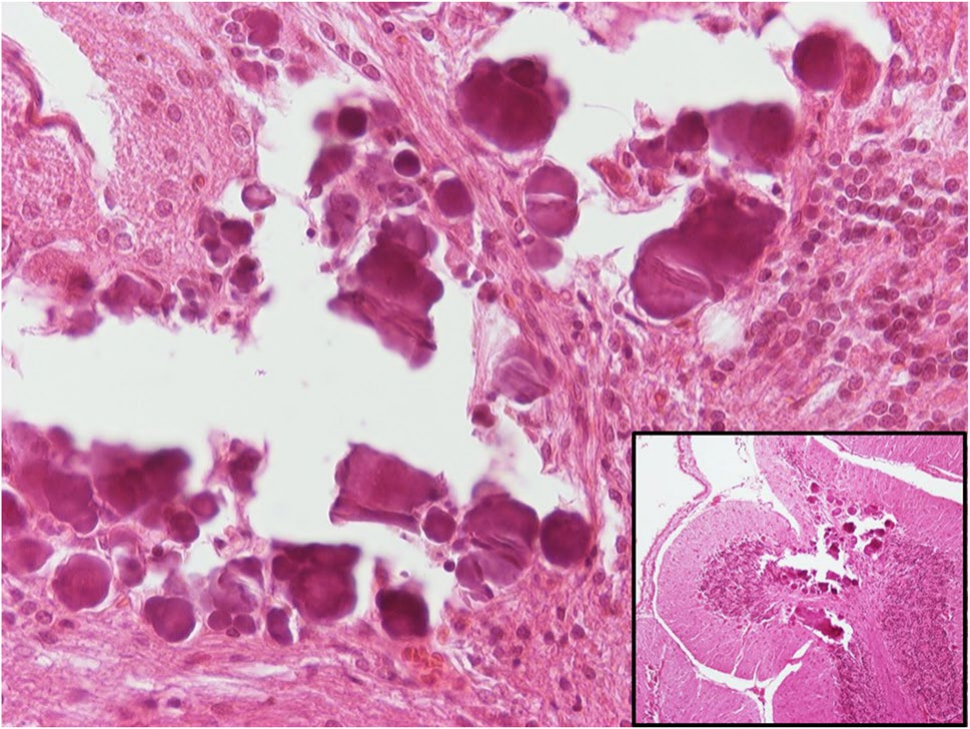
H/E × 400 (insert H/E × 100), cerebellum from group receiving NPs without chitosan: dystrophic microcalcifications, possibly after hemorrhage

From the same five animals of this subgroup, a different one from the immediately mentioned above animal showed additionally dystrophic microcalcifications at the cerebellum, possibly after hemorrhage (Fig. 11), though this may be an incidental finding.

One non-quantifiable finding was the presence of many capillary vessels with erythrocytes in rouleaux formation. This was observed in both groups, though it was more prominent in the samples from the group that received NPPs.

#### Statistical analysis of the in vivo testing results

For all animals in this study, the results of the histopathology examination for each organ were added to have the score for each animal (Table 2). The score from the subgroup of animals that received NPCs and was sacrificed 24 h after administration showed no difference when compared with the score from the control group, negating the value of a *t*-test. From the scores of the other subgroup, a *t*-test comparison with the control showed *P* = 0.10, higher than 0.05; thus, there is no statistically significant difference between the two.

As discussed above, the two subgroups that received NPPs provided more histological findings, both in severity and number of involved animals. Performing a *t*-test between the scores from the subgroup that was sacrificed 24 h after the administration, a *P* = 0.06 was found. This is very close, but still higher than 0.05. However, in the subgroup, that was sacrificed 1 week after the administration, the *t*-test for its results and those from control showed a *P* = 0.002, lower than 0.05. This means that the difference of findings is statistically significant.

## Discussion

The results from the MTT testing show that the NPCs have better results than NPPs, as the former present viability of 90% and above, a clear sign that those NPs are biocompatible. The percentage of cell viability for the NPPs is below or slightly above that the cut-off we had for biocompatibility. In other similar studies [16], a viability between 60 and 90% indicated that the examined material is slightly cytotoxic.

Another observation from the MTT testing is that for both types of NPs, the percentage of cell survival in comparison to the control was increasing for every day of testing. This can be explained by taking into consideration the drug-release/biodegradation study results, where around 50% of the NPs had biodegraded within the first 24 h. Thus, as days progress, there are fewer NPs interacting with the cells in the wells.

The results from the in vivo testing are also in favor of NPCs.

There were no abnormal findings in the samples from the stomach and lungs, and the findings from the kidneys and liver samples are mild and may be considered as incidental. The findings from the skin and subcutaneous tissue from the injection area show more intense inflammatory reaction in the animals that received NPPs.

The elephant in the room is obviously the findings from the brain samples. In the group that received NPPs, and in specific in the subgroup sacrificed 1 week after the administration, there were many animals with irreversible cell damage, including damage to the neurons. The type of damage and especially its location, as well as the effects on endothelial cells, indicate damage from hypoxia [15, 17].

The reason why only the brain is affected has to do not only with its vulnerability to hypoxia, as brain cannot produce adenosine triphosphate (ATP) anaerobically [17], but because that the brain capillaries differ from those in other organs. These differences include the lack of fenestrations and presence of tight junctions, as well as extremely low transcytosis activity compared to capillaries from the periphery. In the brain, the endothelial cells, pericytes, and basement membrane proteins, as well as the end-feet of astrocytes, form the blood-brain barrier (BBB) [18]. Although small molecules may easily cross the BBB via several pathways [19], under normal conditions, the BBB prevents the crossing of most molecules [20].

Nanotechnology offers new approaches for crossing the BBB [19]. In specific, NPs have several properties that enable them to cross the BBB, including their small size and surface current potential [21]. Chitosan is a cationic polymer and when used as coating of NPs increases their permeability through the cellular membrane, which is negatively charged [7, 22]. As shown in our biodegradation studies, NPs without chitosan coating tend to self-organize in clusters that may lead into the formation of aggregations. These aggregations, though not large enough to act as emboli, affect the potential toxicity of NPs, by altering their molecular interactions with various proteins and other macromolecules [23]. It should be noted that particles larger than 300 nm can be eliminated from the human body by resident macrophages [24]. Our hypothesis is that the endothelial cells of the BBB are affected by the increased toxicity of NPPs in their capacity to effectively provide the brain with oxygen. This is supported by the fact that the brain lesions were compatible with acute ischemia and the changes in the endothelial cells and perivascular edema that were observed in our study. This is not at all the case when the NPs are created with the addition of a cationic polymer, like chitosan, that biodegrade without forming aggregates [9] and thus without affecting the endothelial cells.

Both NP types were loaded with curcumin, a drug with antioxidant and anti-inflammatory properties [1], and this may explain why the inflammatory response was mild.

As NPs have shown great potential for use in managing brain disorders, their possible neurotoxicity has been examined in the literature, and several mechanisms of induction are being proposed [25]. To the best of our knowledge, this is the first study where there is histopathological evidence of acute brain ischemia after administration of PLGA NPs without coating. The importance of this finding is increased by the fact that nanomedicines are proposed for treatment of ischemic brain stroke. In a review paper for this subject, the studies that included in vivo testing with PLGA NPs used them with polymeric coating [26]. There is a recent study where PLGA NPs are proposed as drug carrier for preventing brain ischemia caused by diabetes mellitus, and the authors did mention a polymeric coating. The authors demonstrate in vivo the neuroprotective effects of PLGA NPs. A serious limitation is the fact that the sampling of the brain specimens was from the forebrain and did not include the oxygen-sensitive areas like the hippocampus, the hypothalamus, and the cerebellum [27]. Our findings agree with those of Chung et al. that there are no lesions in the forebrain. On the other hand, there were lesions at the hippocampus, middle brain, and cerebellum, which were small and focal. Thus, important findings are easy to be missed, highlighting the role of Pathology in research.

Our study agrees with the established literature, where is suggested for reduced toxicity of NPs not only to use those that are not only created from biocompatible materials but also to utilize coating or shell and to link them with an antioxidant factor [28]. This is reflected at both in vitro and in vivo testing for the PLGA NPs, coated with chitosan that carry curcumin.

## Current limitations and future research steps

The authors would like to clarify that potential contamination was considered when reviewing the results, and we used data from unpublished in vivo experiments with similar methodology, and these findings are in line with those herein presented. In addition, all tasks (characterization, biodegradation, MTT testing, in vivo testing) were performed using NPs created with the same methodology but in different batches, and all results correlate towards the same outcome. So, the possibility of contamination is very small.

As described above, the results of the in vivo testing of the subgroup that received NPCs and was sacrificed at 24 h did not allow for statistical significance testing. Via the G* Power 3.1 software, but this time using the data from the current study, it was shown that to have statistically significant findings, the subgroup size should be 26 animals. However, before repeating animal testing, several items should be re-addressed, like administration way, drug load, dosage, and drug bioavailability at the target organ.

## Conclusion

PLGA NPs with chitosan coating are superior in terms of potential toxicity when compared to PLGA NPs without chitosan coating, as the former show excellent results both for the in vitro and in vivo toxicity testing. Thus, they have the potential to be used as drug carriers. The inclusion of Pathology in cutting-edge research is multiplier for its efficiency, as even minor details, that may otherwise be missed, may impact the clinical implementation of nanomedicines.

## Abbreviations

PLGA: Poly- (lactic-co-glycolic acid)
NP: Nanoparticle
COVID-19: Coronavirus disease 2019
PLA: Poly lactic acid
PGA: Glycolic acid
US FDA: United States Food and Drug Administration
μm: Micrometer
ml: Milliliter
PVA: Polyvinyl alcohol
LTFN: Laboratory of Thin Films, Nanobiomaterials–Nanosystems and Nanometrology
AUTH: Aristotle University of Thessaloniki
NPP: PLGA nanoparticle without chitosan coating (plain)
NPC: PLGA nanoparticle with chitosan coating
LD: Lethal dose
Kg: Kilogram
H/E: Hematoxylin/eosin
PBS: Phosphate-buffered saline
ATP: Adenosine triphosphate
BBB: Blood-brain barrier
